# Metal-Biosurfactant Complexes Characterization: Binding, Self-Assembly and Interaction with Bovine Serum Albumin

**DOI:** 10.3390/ijms20122864

**Published:** 2019-06-12

**Authors:** Tomasz Janek, Lígia R. Rodrigues, Eduardo J. Gudiña, Żaneta Czyżnikowska

**Affiliations:** 1 Department of Biotechnology and Food Microbiology, Wrocław University of Environmental and Life Sciences, 51-630 Wrocław, Poland; 2 Centre of Biological Engineering, University of Minho, 4710-057 Braga, Portugal; lrmr@deb.uminho.pt (L.R.R.); egudina@deb.uminho.pt (E.J.G.); 3 Department of Inorganic Chemistry, Faculty of Pharmacy, Wroclaw Medical University, 50-556 Wrocław, Poland; zaneta.czyznikowska@gmail.com

**Keywords:** biosurfactant, lipopeptides, molecular modeling, fluorescence quenching, BSA, divalent counterions

## Abstract

Studies on the specific and nonspecific interactions of biosurfactants with proteins are broadly relevant given the potential applications of biosurfactant/protein systems in pharmaceutics and cosmetics. The aim of this study was to evaluate the interactions of divalent counterions with the biomolecular anionic biosurfactant surfactin-C_15_ through molecular modeling, surface tension and dynamic light scattering (DLS), with a specific focus on its effects on biotherapeutic formulations. The conformational analysis based on a semi-empirical approach revealed that Cu^2+^ ions can be coordinated by three amide nitrogens belonging to the surfactin-C_15_ cycle and one oxygen atom of the aspartic acid from the side chain of the lipopeptide. Backbone oxygen atoms mainly involve Zn^2+^, Ca^2+^ and Mg^2+^. Subsequently, the interactions between metal-coordinated lipopeptide complexes and bovine serum albumin (BSA) were extensively investigated by fluorescence spectroscopy and molecular docking analysis. Fluorescence results showed that metal-lipopeptide complexes interact with BSA through a static quenching mechanism. Molecular docking results indicate that the metal-lipopeptide complexes are stabilized by hydrogen bonding and van der Waals forces. The biosurfactant-protein interaction properties herein described are of significance for metal-based drug discovery hypothesizing that the association of divalent metal ions with surfactin allows its interaction with bacteria, fungi and cancer cell membranes with effects that are similar to those of the cationic peptide antibiotics.

## 1. Introduction

Lipopeptide biosurfactants are surface-active biomolecules that are produced by a variety of microorganisms [1]. These compounds gained the attention of microbiologists, chemists and biochemists due to their high biodiversity, but also to their activity, low toxicity and good biodegradability in comparison to their synthetic counterparts [2]. Recent studies highlighted a number of interesting biological and chemical properties of biosurfactants and many pharmaceutical and medical applications have been suggested [3,4,5]. In particular, their ability to disturb the integrity of cell membranes of bacteria, yeasts and tumor cells, leading to metabolites leakage and ultimately to cell lysis; as well as their propensity to partition at the interfaces, modifying surface properties and thus affecting microorganisms adhesion, which are important functions for antimicrobial and anti-biofilm applications [6,7].

**Figure 1:**
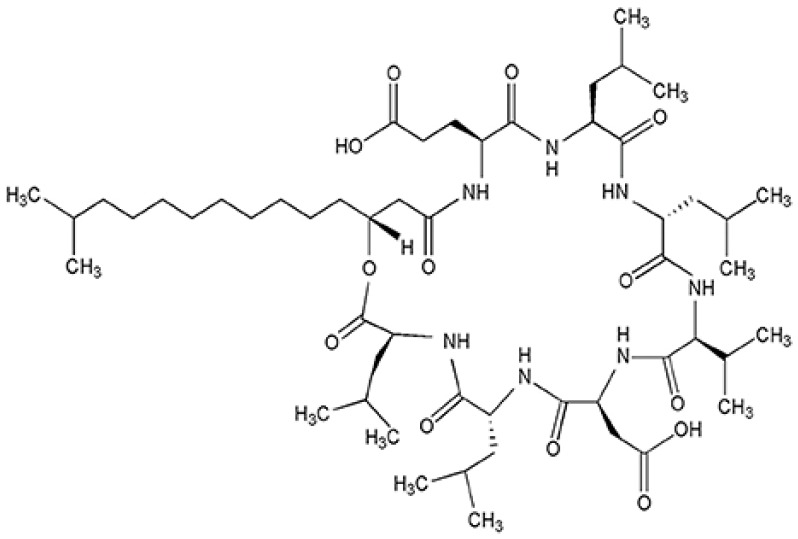
Chemical structure of surfactin-C_15_. Seven amino acids are arranged in the cyclic ring connected with a β-hydroxyl fatty acid with a chain length of fifteen carbon atoms to form a cyclic lactone ring.

Surfactin is a cyclic lipopeptide biosurfactant, produced by various strains of *Bacillus subtilis* [8,9]. Surfactin consists of a heptapeptide head group with the sequence Glu-Leu-d-Leu-Val-Asp-d-Leu-Leu closed to a lactone ring by a β-hydroxyl fatty acid (Figure 1). Surfactin exhibits significant biological activities [10]. For example, it shows potent antimicrobial and anti-adhesive action against several pathogenic microorganisms on medical implants [11,12] as well as antitumor activities.

The chemistry of metal coordination surfactants is nowadays becoming popular, particularly in the design of drugs exhibiting improved biological activity [13,14]. The potential application of cationic double-chained metallosurfactants in medicine has been studied over the past few years [15]. Several metal chelates are known to exhibit anticancer, antiviral, antibacterial, and antifungal activities [16,17]. In several cases, the metal coordination surfactants have been found to exhibit better antimicrobial activity than the chelating agents themselves [16].

Interactions of proteins with surfactants have been extensively studied for the past few years due to their numerous applications in biological and industrial systems [18,19,20]. Investigating the interaction of biological active compounds with albumins may provide useful information on the structural features of drug-protein complexes that determine the therapeutic effectiveness of drugs [21,22,23]. Serum albumins are the most abundant proteins in blood plasma and the circulatory system and are responsible for binding a wide variety of metal ions, drugs, fatty acids, and surfactants [24]. Thus, the study of the binding of probes or drugs with albumins becomes a relevant research field in chemistry, life sciences, and clinical medicine [19,25]. Several biophysical methods such as surface tension analysis, fluorescence and circular dichroism (CD) spectroscopy, and molecular docking have been used to unravel the interactions between surfactants and proteins [18,20].

In this work, the biomolecular interactions between divalent counterions and surfactin-C_15_ were studied by conformational analysis using semi-empirical methods, changes in surface tension and dynamic light scattering (DLS). Subsequently, the interactions between surfactin-C_15_ and metal-surfactin complexes with BSA were studied by fluorescence spectroscopy. In addition, molecular docking was used to better understand the interaction between surfactin-C_15_ and metal-surfactin complexes with BSA. This study provides a molecular basis for the applications of surfactin/BSA and metal-surfactin/BSA complexes in biological, pharmaceutical, and medical systems.

## 2. Results and Discussion

### 2.1. Conformational Analysis

The conformational analysis was performed to get an insight in the molecular structure of metal-lipopeptide complexes and to determine the impact of ions binding to the conformation of surfactin. Moreover, the results obtained became the starting point for further investigation concerning the self-aggregation ability of the lipopeptide after complexation and molecular docking. The data are presented in the Section 2.2 and Section 2.4.

Our previous studies [26,27] have shown that Cu^2+^ ions preferentially bind to amide nitrogens, whereas the remaining divalent cations (Zn^2+^, Mg^2+^ and Ca^2+^) are associated with the oxygen atoms of lipopeptide biosurfactants. Additionally, Gang et al. [28] reported that based on their molecular dynamic simulations, surfactin exhibits high binding affinity to calcium counterions and both carboxyl groups present in its structure are accessible for metals.

**Figure 2:**
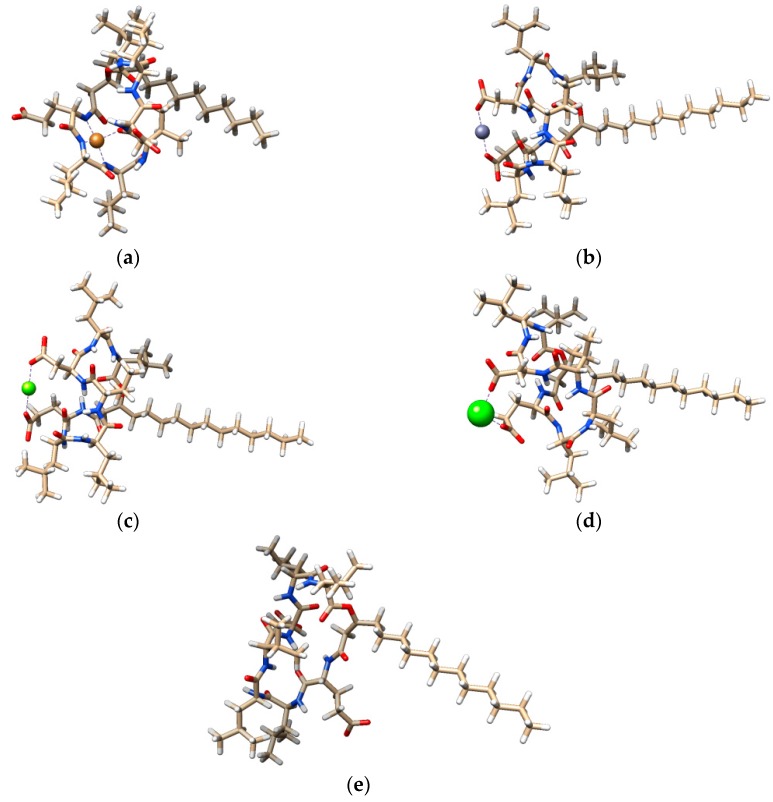
Predicted orientations of the lowest energy conformations of surfactin-C_15_ and its complexes with divalent metal ions: Cu^2+^-surfactin (**a**), Zn^2+^-surfactin (**b**), Mg^2+^-surfactin (**c**), Ca^2+^-surfactin (**d**) and surfactin (**e**).

In the present study, the metal coordinated geometries of surfactin-C_15_ were examined at PM6 level of theory considering our experimental findings. The lowest energy conformers of analyzed complexes are represented in Figure 2.

As can be observed from Figure 2, the simulated structures reveal that the oxygen atoms belonging to the carboxyl groups of aspartic and glutamic acid are important binding sites for Mg^2+^, Zn^2+^ and Ca^2+^ ions. Contrary to the results obtained for pseudofactin, the main chain carbonyl oxygen atoms were not involved in coordination [26]. Additionally, we were not able to obtain the four-coordinated complex for zinc. Similar to our earlier findings, three amide nitrogens from the main chain of surfactin-C_15_ ring can coordinate the Cu^2+^ ions in almost planar configuration. Furthermore, in complex formation the oxygen atom from the lipopeptide ring is involved. Additionally, the stabilization of some complexes was enhanced by the presence of hydrogen bonds. Finally, complex formation led to significant changes in the structure of the lipopeptide, the most important of which are recorded in the case of zinc and copper complex formation. The comparison of the calculated structures is presented in the Supplementary information (Figure S1).

### 2.2. Divalent Counterions Effects on Surface Tension 

**Figure 3:**
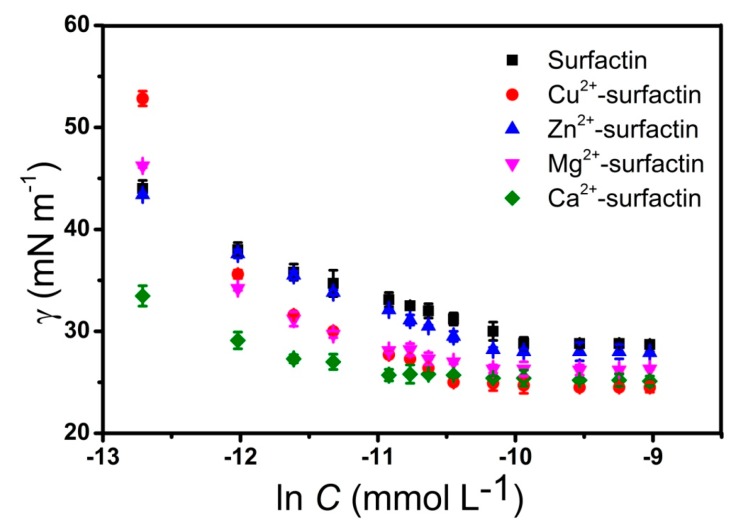
Surface tension profiles of surfactin and metal-surfactin complexes in Hepes buffer (pH 7.4) at 25 °C. The values represent the mean of triplicates ± SD.

The surface tension curves of surfactin-C_15_ at different concentrations of Cu^2+^, Zn^2+^, Mg^2+^ and Ca^2+^ are shown in Figure 3. For the pure surfactin in the Hepes buffer without divalent counterions, the critical micelle concentration (CMC) value was 0.045 mM. Regarding the effect of divalent cations, Ca^2+^ decreased the CMC value of surfactin-C_15_ more than the other cations. Compared with the surfactin-C_15_ solution without metal ions, the CMC of Ca^2+^-surfactin decreased from 0.045 mM to 0.017 mM. The addition of 0.1 mM of Cu^2+^, Mg^2+^ and Zn^2+^ to surfactin-C_15_ reduced the CMCs to 0.033 mM, 0.036 mM and 0.038 mM, respectively. All the tested metal ions led to reductions of the surface tension of surfactin-C_15_ at the CMC (γ_cmc_), being the highest reductions obtained with Cu^2+^ and Ca^2+^ (from 28.4 mN·m^−1^ up to 24.5 and 25.1 mN·m^−1^, respectively). The peptide ring of surfactin adopts a “horsesaddle” structure in solution, with the two charged residues forming a “claw”, which is a potential binding site for divalent cations [29].

As it was reported in earlier studies, divalent cations can significantly influence the properties of micellar systems [30,31]. For example, it is known that the addition of metal ions decreases the CMC of surfactin. This is probably related with two important competing forces during the micellization process. Electrostatic interactions, which are responsible for the repulsion of hydrophilic groups of the lipopeptide inhibit the formation of micelles. On the other hand, hydrophobic interactions are responsible for initiation of micellization through exclusion of fatty acid chains from the aqueous environment. The presence of divalent cations additionally supports the self-assembly process. From the data shown in Table 1, it can be concluded that metal ions binding significantly affects the molecular volume of surfactin complexes. In the case of the Cu^2+^-surfactin system, *V*_mic_^PM6^ is twice lower than in the case of the free lipopeptide. Such big differences influence the aggregation number. Generally, *N*_agg_ decreases after binding of cations. The experimental micelle volumes and hydrodynamic radii with their theoretically calculated counterparts and aggregation numbers are shown in Table 1. The experimental (*R*_H_^DLS^) and theoretically calculated (*R*_H_^PM6^) hydrodynamic radii of surfactin-C_15_ were 2.46 nm and 2.40 nm, respectively. Addition of Cu^2+^, Zn^2+^, Mg^2+^ and Ca^2+^ reduces the size of the microstructures. In this study, we observed the largest modifications of surfactin-C_15_ conformation due to the binding of Cu^2+^; in that case *R*_H_^DLS^ and *R*_H_^PM6^ were 1.82 nm and 1.80 nm, respectively. The data obtained from the semiempirical study are in agreement with DLS spectroscopy results (Table 1).

### 2.3. Fluorescence Measurements

**Figure 4:**
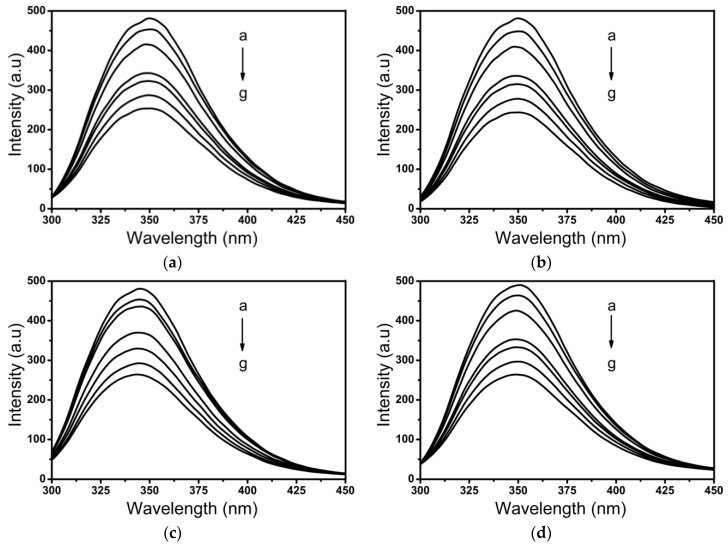

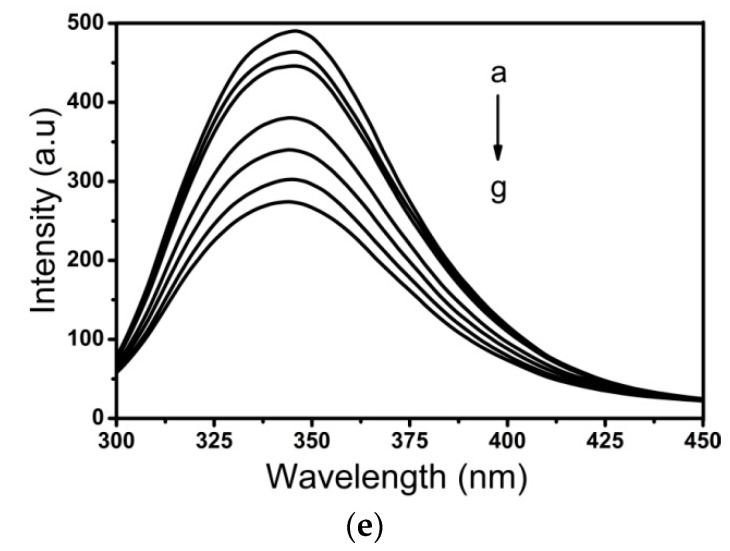
Fluorescence emission spectra of bovine serum albumin (BSA) with different concentrations of Cu^2+^-surfactin (**a**), Zn^2+^-surfactin (**b**), Mg^2+^-surfactin (**c**), Ca^2+^-surfactin (**d**) and surfactin (**e**). Conditions: BSA: 15 µM; metal-lipopeptide (a–g): 0, 10, 20, 50, 70, 100 and 120 µM; pH = 7.4; *T* =25 °C.

Fluorescence spectroscopy is a valuable tool for studying ligand-protein interactions. BSA contains two tryptophan residues, Trp134 and Trp213, which are located in sub-domain IB and sub-domain IIA, respectively [32]. The effect of surfactin and metal-surfactin complexes on intrinsic fluorescence of BSA is shown in Figure 4. The maximum emission wavelength of BSA was found at 348 nm, and the intensity decreased regularly upon increasing the concentration of surfactin and metal-surfactin complexes. This interaction takes place adjacent to the Trp in BSA and changes the polarity around the fluorophore. This phenomenon is ascribed to the formation of surfactin/BSA and metal-surfactin/BSA complexes [33].

**Figure 5:**
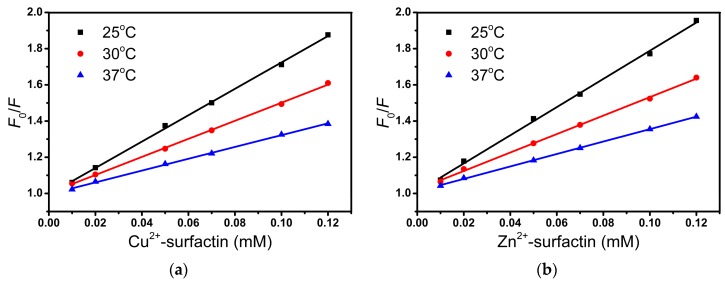

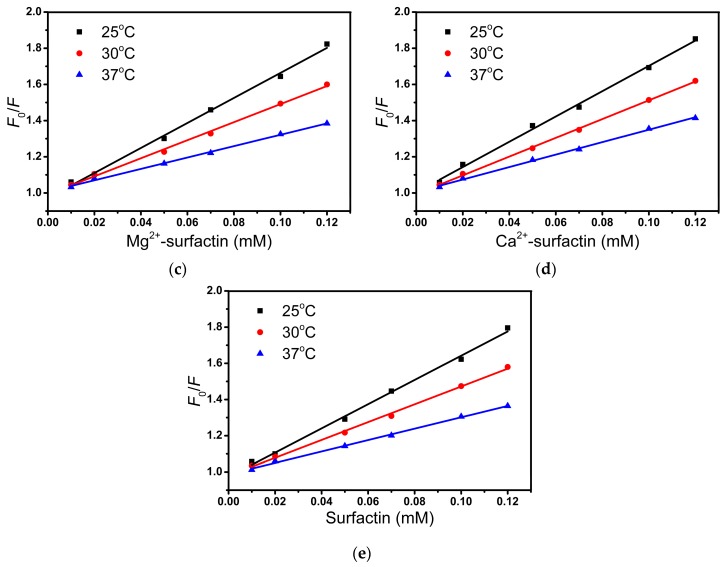
Stern–Volmer plots of the fluorescence titration of Cu^2+^-surfactin (**a**), Zn^2+^-surfactin (**b**), Mg^2+^-surfactin (**c**), Ca^2+^-surfactin (**d**) and surfactin (**e**) with BSA. Conditions: BSA: 15 µM; pH = 7.4; *T* = 25/30/37 °C.

Fluorescence quenching can occur through dynamic and static mechanisms. Both dynamic and static quenching require molecular contact between the fluorophore and the quencher. The Stern–Volmer quenching (*K*_SV_) values decrease with an increase in temperature for static quenching, but the reverse effect is observed for dynamic quenching. Quenching parameters were calculated from the Stern–Volmer equation [34]:

(1) $$F_{0}/F=1+K_{SV}\left[Q\right]=1+k_{q}\tau_{0}\left[Q\right]$$

where *F*_0_ and *F* are the fluorescence intensities in the absence and presence of surfactin or metal-surfactin complexes, respectively; *K*_SV_ is the Stern–Volmer quenching constant; [Q] is the concentration of surfactin or metal-surfactin complexes; *k*_q_ is the biomolecular quenching rate constant; and τ_0_ is the lifetime of the fluorophore. The fluorescence lifetime of BSA is about 5 ns [35]. The *K*_SV_ values obtained from the plot of [Q] versus *F*_0_/*F* (shown in Figure 5) were in the range from 6.696 × 10^3^ to 7.787 × 10^3^ M^−1^ corresponding to the surfactin and metal-surfactin complexes. The *K*_SV_ values obtained from the Stern–Volmer plot at three different temperatures (25, 30, and 37 °C) are presented in Table 2. We observed that for the surfactin/BSA and metal-surfactin/BSA systems, the *k*_q_ values range from 6.294 × 10^11^ to 1.557 × 10^12^ M^−1^·s^−1^ and are greater than the maximum collision quenching constant: 2 × 10^10^ M^−1^·s^−1^ [36]. This suggests that the formation of the surfactin/BSA and metal-surfactin/BSA complexes occurred through a static quenching.

**Figure 6:**
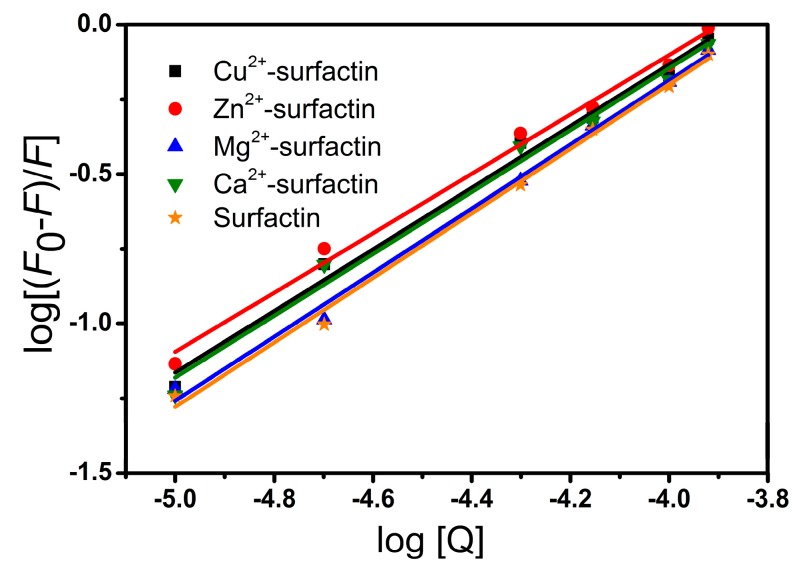
Plots of log[(*F*_0_−F)/F] versus log [Q] for surfactin- and metal-surfactin complexes-BSA binding; pH = 7.4; *T* =25 °C.

For the static quenching interaction, the binding constant (*K*_b_) and the number of binding sites (*n*) can be determined by the following equation:

(2) $$\log\left[\left(F_{0}-F\right)/F\right]=\log K_{b}+n\log\left[Q\right]$$

where *F*_0_ and *F* are the fluorescence intensities in the absence and presence of surfactin or metal-surfactin complexes, respectively, and [Q] is the concentration of surfactin or metal-surfactin complexes. Binding constants obtained from the plot of log [Q] versus log [(*F*_0_−*F*)/*F*] (Figure 6) corresponding to surfactin and metal-surfactin complexes ranged from 0.746 × 10^4^ to 1.315 × 10^4^ M^−1^. The binding parameters are summarized in Table 3. Surfactin exhibits more efficient binding properties than the metal-surfactin complexes. Regarding the binding constants (*K*_b_), surfactin has values higher than all the tested metal-surfactin complexes. The value of *n* represents the number of binding sites, and it is close to one in our experiment, which might suggest that the tryptophan in BSA is accessible to surfactin and metal-surfactin complexes. The above results are similar to those reported for metal-amphisin/BSA [27].

Thermodynamic parameters for a binding interaction can be used as major evidence for the nature of intermolecular forces. There are four types of interaction forces, which could play a role in ligand binding to proteins: Van der Waals forces, hydrophobic forces, electrostatic interactions and hydrogen bonds [37]. Thermodynamic parameters relying on temperatures were analyzed to characterize the acting force between surfactin and metal-surfactin complexes with BSA. To obtain such information, the thermodynamic parameters were calculated from the Van’t Hoff relation [37]:

(3) $$\ln K=-\frac{\Delta H^{0}}{RT}+\frac{\Delta S^{0}}{R}$$

(4) $$\Delta G^{0}=\Delta H^{0}-T\Delta S^{0}=-RT\ln K$$

where *K* is the binding constant, *R* is the gas constant and *T* is the experimental temperature. Δ*H* denotes the enthalpy change and Δ*S* denotes the entropy change of the binding process. The Δ*H* and Δ*S* values were calculated from the slope and intercept of the Van’t Hoff plot (Figure S2), respectively. The values of the thermodynamic parameters are shown in Table 4. The negative sign for Δ*G* means that the interaction process is spontaneous. The negative Δ*H* and Δ*S* values showed that both hydrogen bonds and the van der Waals interaction played major roles in the binding of surfactin and metal-surfactin complexes to BSA. This observation is supported by the docking results.

### 2.4. Molecular Docking Study

**Figure 7:**
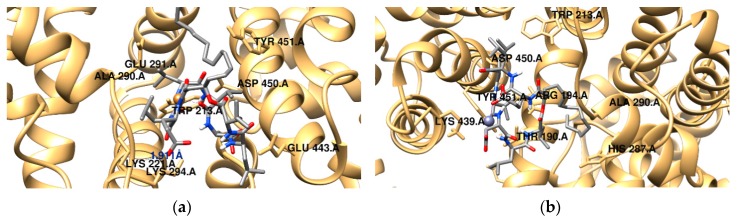

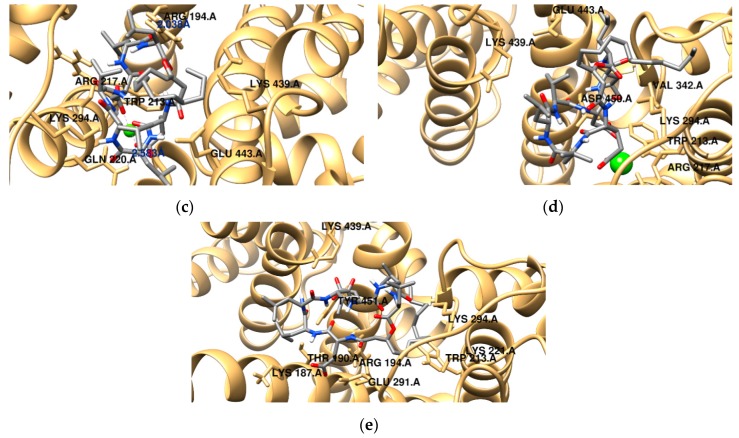
Predicted binding modes of surfactin and its metal complexes with BSA using molecular docking simulations. Cu^2+^-surfactin/BSA (**a**), Zn^2+^-surfactin/BSA (**b**), Mg^2+^-surfactin/BSA (**c**), Ca^2+^-surfactin/BSA (**d**), and surfactin/BSA (**e**).

BSA contains two major drug binding sites in the sub-domains IIA and IIIA: Tryptophan residues Trp134 and Trp213, being Trp213 located in the hydrophobic binding pocket of the protein. In order to predict the binding mode of surfactin and the metal-surfactin complexes to BSA, the docking simulations were performed and binding forces were discussed. Figure 7 and Figure S3 show the docking results for the different compounds. The obtained data indicate that all the surfactin complexes studied can bind to BSA.

The binding and geometrical orientation of analyzed systems strongly depend on the presence of divalent metal ions. Docking results revealed that the van der Waals forces play a significant role in the stabilization of the protein-surfactant complexes and electrostatic terms are negligible. For example, for the system with the free lipopeptide, the sum of *ΔG*_vdw_, *ΔG*_hbond_ and *ΔG*_desolv_ is about −46 kJ·mol^−1^, whereas the value of electrostatic energy is −0.3 kJ·mol^−1^. According to the data, Ca^2+^-surfactin/BSA complex is characterized by the lowest binding free energy (−42 kJ·mol^−1^). All the compounds studied are located in close proximity of Trp213, but the arrangement of the hydrophilic and hydrophobic domains of the lipopeptide in the binding site differs significantly. The surfactin-C_15_ molecule is mainly surrounded by polar amino acids (Thr190, Gln220, Tyr451, Cys447) and charged amino acids (such as Lys187, Arg194, Lys221, Glu291, Lys294, Lys439). It can be also observed that the Mg^2+^-surfactant complex is located in the hydrophobic cavity created by Pro338, Tyr340, Pro440 and Pro446 residues. In addition, the amino acids Arg194, Arg217, Lys294 and Lys439 are in the close proximity of the ligand. A hydrogen bond is found involving the H–N- group of Arg194 and the lipopeptide oxygen atom (2.04 Å). The second hydrogen bond is formed between the oxygen atom of the side chain of Glu of surfactin-C_15_ and the H–N- group of Gln220. Cu^2+^-surfactin complex is surrounded by a number of amino acid residues, namely Trp213, Lys221, Ala290, Glu291, Lys294, Asp450 and Tyr 451. There is also a possibility of H-bonding interaction involving the carboxyl group of Asp from surfactin and Lys294 from the protein, showing a distance 1.9 Å (Figure 7a). The intermolecular interaction energies estimated during the molecular docking procedure for the complex in question revealed that binding between lipopeptide and BSA occurs mainly through hydrogen bonding, van der Waals and hydrophobic interactions, which supports the experimental results ((*ΔG*_vdw_ + *ΔG*_hbond_ + *ΔG*_desolv_) = −71 kJ·mol^−1^). In the case of Zn^2+^-surfactin complex (Figure 7b), the polar head group of surfactin is exposed towards polar and charged amino acid residues (Trp213, Tyr451, Asp450 and Lys439), while the hydrophobic tail is observed to be encompassed by a number of polar amino acid residues, namely, Thr190, His287. The binding free energy of Zn^2+^-surfactin/BSA complex was established as −40 kJ·mol^−1^.

## 3. Materials and Methods

### 3.1. Surfactin-C_15_ Production and Purification

The bacterial strain *Bacillus subtilis* #309 previously isolated from a crude oil sample obtained from a Brazilian oil field [38] was used in this study. The strain #309 was stored at −80 °C as a glycerol stock in the Department of Biotechnology and Food Microbiology, Wrocław University of Environmental and Life Sciences, Wrocław, Poland. The production of surfactin by *B. subtilis* #309 was performed in 1000 mL flasks containing 200 mL of a culture medium with the following composition: 10 g·L^−1^ of NaCl (POCH, Gliwice, Poland), 10 g·L^−1^ of sucrose (POCH), 2 g·L^−1^ of NH_4_NO_3_ (Chempur, Poland), 5 g·L^−1^ of Na_2_HPO_4_ (POCH), 2 g·L^−1^ of KH_2_PO_4_ (POCH), and 0.2 g·L^−1^ of MgSO_4_ × 7H_2_O; pH 7.0. Each flask was inoculated with 1% (*v*/*v*) of a pre-culture of *B. subtilis* #309 grown in LB medium (10 g·L^−1^ NaCl; 10 g·L^−1^ tryptone; 5 g·L^−1^ yeast extract; pH 7; A&A Biotechnology, Gdynia, Poland) at 37 °C and 180 rpm for 24 h. The flasks were incubated at the same conditions for 24 h. At the end of the incubation period, the cultures were centrifuged (10,000× *g*, 15 min), and the cell-free supernatant was used to recover the biosurfactants produced through acid precipitation. Briefly, the supernatant was adjusted to pH 2.0 with 6 M HCl and left overnight at 4 °C. Afterwards, the precipitate was collected by centrifugation (10,000× *g*, 15 min) and washed twice with acidified water (pH 2.0). The precipitated biosurfactants were dissolved in demineralized water and the pH was adjusted to 7.0 using 1 M NaOH. Subsequently, the biosurfactants present in the crude mixture were purified through reversed-phase high-performance liquid chromatography (RP-HPLC) using a Waters 600 HPLC system (Waters, Milford, MA, USA) equipped with an Xterra Prep RP18 OBD column (5 μm, 18 × 100 mm; Waters). The solvent system consisted of solvent A (i.e., 0.1% aqueous trifluoroacetic acid) and solvent B (i.e., 0.1% trifluoroacetic acid in acetonitrile). The surfactin analogs were eluted at a flow rate of 4 mL min^−1^ with the following 40-min gradient (% A:B *v*/*v*): injection start (30:70), 5 min (30:70), 10 min (20:80), 20 min (20:80), 21 min (0:100), 31 min (0:100), 32 min (30:70), and 40 min (30:70). Mass spectrometry of the purified surfactin-C_15_ revealed a purity greater than 99% (Figure S4).

### 3.2. Surface Tension Measurements

The surface tension (γ) measurements were performed at room temperature (25 °C) according to the du Noüy’s ring method [39] using a KRÜSS K20 Tensiometer (KRÜSS GmbH, Hamburg, Germany). Surfactin-C_15_ and the different metal ions were dissolved in a 10 mM Hepes (Sigma-Aldrich, St. Louis, MO, USA) buffer (pH 7.4) and mixed to obtain several mixtures containing a constant metal ions concentration (0.1 mM) while the surfactin-C_15_ concentration varied from 0.003 to 0.12 mM. Ultrapure water was used to calibrate the tensiometer. The platinum ring was thoroughly cleaned with Millipore water between the different measurements. All measurements were performed in triplicate. The surface tension of the control sample (10 mM Hepes (pH 7.4)) was 69.4 mN·m^−1^. The surface tension data were analyzed using the Origin software provided with the equipment to obtain the CMC values.

### 3.3. Micelles Size Measurement by Dynamic Light Scattering (DLS)

The size of the micelles formed by surfactin-C_15_ diluted in 10 mM Hepes buffer (pH 7.4) in the presence of different concentrations of metal ions was examined through DLS using Zetasizer Nano-ZS spectrometer (Malvern, Worcestershire, UK). The particle size estimations were made at fixed 173° backscattered angle, and the experimental temperature was maintained at 25 °C. All measurements were performed in triplicate.

### 3.4. Fluorescence Measurements

The fluorescence quenching spectra were obtained with a Varian Cary eclipse fluorescence spectrophotometer. All fluorescence spectra were collected using a 1.0-cm quartz cuvette with both excitation and emission band-widths of 5 nm. The interior fluorescence spectra of BSA were scanned over the wavelength range of 300–450 nm with a fixed excitation wavelength at 280 nm. To eliminate the inner filter, the corrected fluorescence was estimated by using equation [40]:

(5) $$F_{cor}=F_{obs}\times 10^{\left(A_{ex}+A_{em}\right)/2}$$

where *F*_cor_ and *F*_obs_ are the corrected and experimentally measured fluorescence intensities, respectively, and *A*_ex_ and *A*_em_ are the measured change in absorbance of the system at excitation and emission wavelengths, respectively.

### 3.5. Molecular Modeling

The conformational analysis of investigated complexes was performed using the Gaussian09 program [41]. The surfactin-metal ion structures were studied based on the PM6 level of theory and optimized geometries were identified as a global minimum on the potential energy surface by harmonic vibrational frequencies calculations. Previous studies performed by Steward et al. [42] demonstrated that this level of theory provides structural parameters in good agreement with experimental data obtained for transition metals biocomplexes. It gives also a good compromise between the accuracy and cost of calculations. In order to consider the solvent effects the polarizable continuum model (PCM) was adopted [43,44,45]. In the present study, we discuss only the lowest energy conformers of the investigated systems. *V*_mon_^PM6^ denotes the molecular volume of surfactin complexes defined by the volume inside a contour of 0.001 electrons/Bohr^3^ density. The measured distance between the most distant atoms was adopted as the micellar radii (*R^PM6^)*. For example, in the case of surfactin, *R^PM6^* was the distance between the farthest carbon atom one of Leu5 and the carbon of the terminal methyl group of β-hydroxydecanoyl fatty acid side chain.

During molecular docking simulations of ligands to BSA we applied the AutoDock 4.2 software [46]. The crystal structure of BSA (PDB code: 3v03) was taken from the Protein Data Bank [47]. Polar hydrogens, Kollman charges and solvent parameters were added to the protein structure and atomic coordinates were stored in a separate file and used as an input. The binding sites were defined using a grid of 80 × 80 × 80 point with a grid space of 0.375 Å. The center of box was located on the binding site of the protein. Lamarckian genetic algorithm with local search was employed with a total of 100 runs for each complex. All calculations included the population of 150 individuals with 27,000 generations and 250,000 energy evaluations. Cluster analysis were performed on docked results using a root mean square (RMS) tolerance of 2.0 Å. The calculated free energy of binding (*ΔG*_binding_) determines the affinity of lipopeptide-BSA complex and can be expressed as:

(6) $$\Delta G_{binding}=\left[\Delta G_{intermolecular}+\Delta G_{internal}+\Delta G_{tors}\right]-\Delta G_{unbound}$$

While the intermolecular interaction energy (*ΔG*_intermolecular_) is the sum of van der Waals, hydrogen bonding, desolvation and electrostatic contribution between the biosurfactant and the protein binding site.

(7) $$\Delta G_{intermolecular}=\left[\Delta G_{vdw}+\Delta G_{hbond}+\Delta G_{desolv}\right]+\Delta G_{el}$$

The UCSF Chimera System was used to visualize the results obtained [48].

## 4. Conclusions

In this work, the interactions between divalent counterions and surfactin-C_15_ were studied through molecular modeling, surface tension, and DLS analysis. For all the tested divalent metal ions, only mononuclear complexes were obtained even when the metal ions were used at a molar ratio of 1:2. Counterions enhanced the surface activity of surfactin-C_15_ and reduced its CMC. The results obtained from fluorescence spectroscopy show that surfactin and metal-surfactin complexes bind with BSA and quench its fluorescence through a static quenching mechanism. On the other hand, surfactin and metal-surfactin complexes bind at the subdomain IIA of BSA through hydrophobic interaction, forming surfactin/BSA and metal-surfactin/BSA complexes. The hydrogen bonds and van der Waals forces play a major role in binding.

## Figures and Tables

**Table 1:** Selected properties of the micellar systems studied. Experimental results represent the mean of 9 replicates ± SD.

**DLS**					
**Micelle Size**	**Surfactin**	**Cu^2+^-Surfactin**	**Zn^2+^-Surfactin**	**Mg^2+^-Surfactin**	**Ca^2+^-Surfactin**
***R*_H_^(DLS)^ (nm)**	2.46 ± 0.03	1.82 ± 0.05	2.05 ± 0.05	2.12 ± 0.07	2.38 ± 0.02
***V*_mic_^(DLS)^ (nm^3^)**	62.36	25.25	36.08	39.91	56.47
**PM6**					
**Micelle Size**	**Surfactin**	**Cu^2+^-Surfactin**	**Zn^2+^-Surfactin**	**Mg^2+^-Surfactin**	**Ca^2+^-Surfactin**
***R*_H_^(PM6)^ (nm)**	2.4	1.8	2	2.05	2.29
***V*_mon_ (nm^3^)**	1.61	1.35	1.46	1.54	1.46
***V*_mic_^(PM6)^ (nm^3^)**	57.9	24.43	33.51	36.09	50.3
***N*_agg_^(PM6)^**	36	18	23	23	34
Vmic(DLS)=43·π·(RH(DLS))3; Vmic(PM6)=43·π·(RH(PM6))3; $N_{agg}=V_{mic}^{\left(PM6\right)}/V_{mon}^{\left(PM6\right)}$					

**Table 2:** Summary of Stern–Volmer data for BSA quenching by surfactin and metal-surfactin complexes.

System	*T* (°C)	*K*_SV_ (M^−1^)	*k*_q_ (M^−1^·s^−1^)	*R* ^2^	SD
Cu^2+^-surfactin	25	7.292 × 10^3^	1.458 × 10^12^	0.999	0.02432
	30	5.102 × 10^3^	1.020 × 10^12^	0.999	0.06321
	37	3.267 × 10^3^	6.534 × 10^11^	0.999	0.07431
Zn^2+^-surfactin	25	7.787 × 10^3^	1.557 × 10^12^	0.997	0.08432
	30	5.186 × 10^3^	1.037 × 10^12^	0.998	0.01275
	37	3.439 × 10^3^	6.878 × 10^11^	0.999	0.06432
Mg^2+^-surfactin	25	6.917 × 10^3^	1.383 × 10^12^	0.997	0.01876
	30	4.973 × 10^3^	9.946 × 10^11^	0.998	0.03871
	37	3.148 × 10^3^	6.296 × 10^11^	0.998	0.07312
Ca^2+^-surfactin	25	6.992 × 10^3^	1.398 × 10^12^	0.997	0.05423
	30	4.993 × 10^3^	9.986 × 10^11^	0.999	0.06531
	37	3.245 × 10^3^	6.490 × 10^11^	0.998	0.01254
Surfactin	25	6.696 × 10^3^	1.339 × 10^12^	0.998	0.03186
	30	4.926 × 10^3^	9.852 × 10^11^	0.998	0.04873
	37	3.147 × 10^3^	6.294 × 10^11^	0.998	0.07126

**Table 3:** Binding constants (*K*_b_) and number of binding sites (*n*) of BSA with surfactin and metal-surfactin complexes at 25 °C.

Complex	*K*_b_ (M^−1^)	*n*	*R* ^2^	SD
Cu^2+^-surfactin	0.980 × 10^4^	1.03	0.995	0.001542
Zn^2+^-surfactin	0.746 × 10^4^	0.99	0.996	0.002106
Mg^2+^-surfactin	1.267 × 10^4^	1.07	0.997	0.001042
Ca^2+^-surfactin	0.986 × 10^4^	1.03	0.996	0.001216
Surfactin	1.315 × 10^4^	1.07	0.997	0.001492

**Table 4:** Thermodynamic parameters for the binding of surfactin and metal-surfactin complexes to BSA.

System	*T* °C	Δ*H* (kJ·mol^−1^)	Δ*S* (J·mol^−1^·K^−1^)	Δ*G*(kJ·mol^−1^)	*R* ^2^	SD
Cu^2+^-surfactin	25	−51.30	−98.23	−22.03	0.997	0.02187
	30			−21.54	0.998	0.01254
	37			−20.85	0.997	0.01047
Zn^2+^-surfactin	25	−51.59	−100.02	−21.78	0.998	0.01784
	30			−21.28	0.998	0.01354
	37			−20.58	0.998	0.01274
Mg^2+^-surfactin	25	−50.42	−95.67	−21.91	0.998	0.01987
	30			−21.43	0.998	0.02487
	37			−20.76	0.999	0.01274
Ca^2+^-surfactin	25	−49.07	−91.10	−21.92	0.999	0.01487
	30			−21.47	0.999	0.02478
	37			−20.83	0.999	0.01147
Surfactin	25	−48.42	−89.20	−21.84	0.998	0.02249
	30			−21.39	0.998	0.01151
	37			−20.77	0.997	0.01876

## Supplementary Materials

Supplementary Materials can be found at https://www.mdpi.com/1422-0067/20/12/2864/s1.

## Abbreviations

Arg	Arginine
Asp	Aspartic acid
BSA	Bovine Serum Albumin
CMC	Critical Micelle Concentration
DLS	Dynamic Light Scattering
Gln	Glutamine
Glu	Glutamic acid
Hepes	4-(2-Hydroxyethyl)-1-piperazineethanesulfonic acid
Leu	Leucine
Lys	Lysine
PCM	Polarizable Continuum Model
PDB	Protein Data Bank
PM6	Parameterization Method 6
Pro	Proline
Thr	Threonine
Trp	Tryptophan
Tyr	Tyrosine
UV	Ultraviolet
Val	Valine
