# Supplementary material for: Metal-Biosurfactant Complexes Characterization: Binding, Self-Assembly and Interaction with Bovine Serum Albumin
Source: Int J Mol Sci. 2019 Jun 12;20(12):2864. doi: 10.3390/ijms20122864 (PMC6627489; doi:10.3390/ijms20122864)
Supplement: Supplementary file 1 [file ijms-20-02864-s001.pdf]

Supplementary Information
Metal-biosurfactant complexes characterization: binding, self-assembly and interaction with bovine serum albumin

Tomasz Janek [1,*], Lígia R. Rodrigues [2], Eduardo J. Gudiña [2] and Żaneta Czyżnikowska [3]

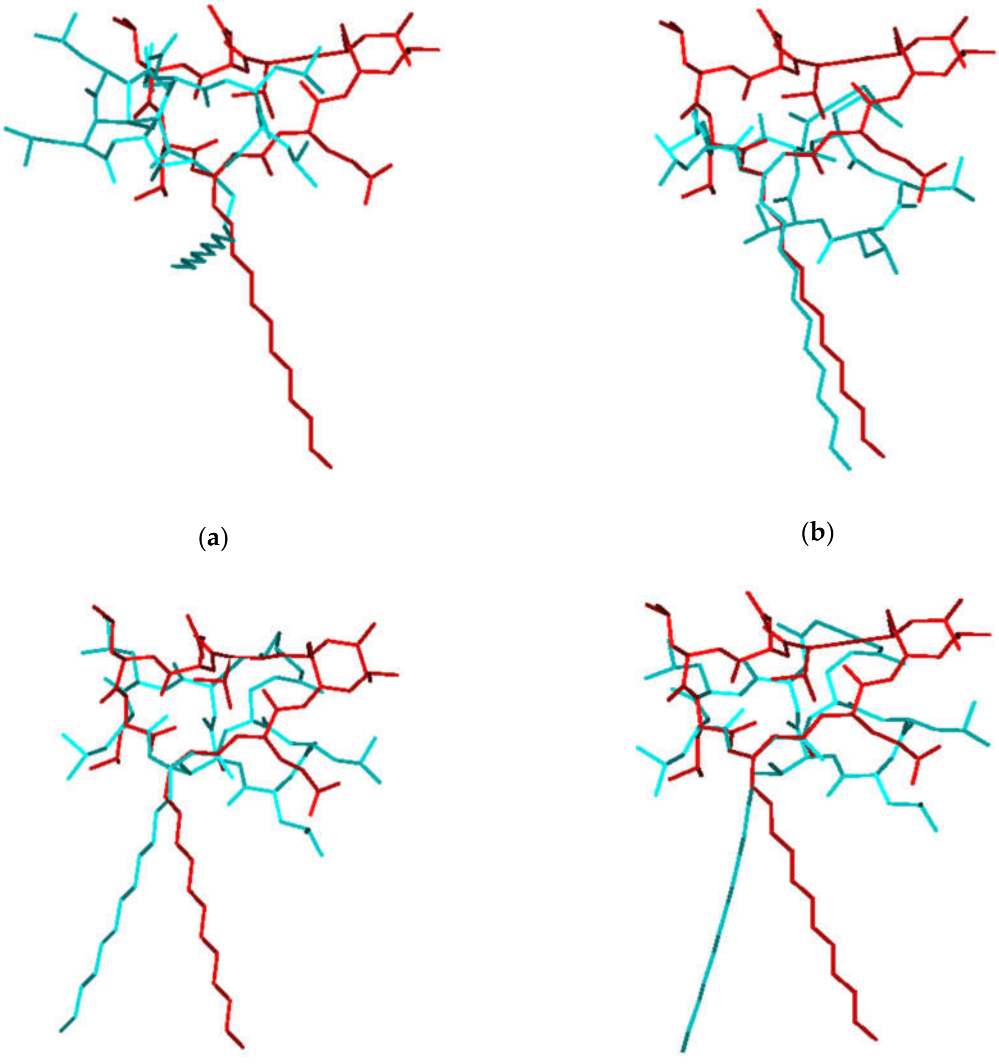

(**a**) (**b**)

(**c**) (**d**)

Figure S1. Comparison of the calculated structures of the surfactin (red) and Cu^{2+}-surfactin (a), Zn^{2+}-surfactin (b), Mg^{2+}-surfactin (c), Ca^{2+}-surfactin (d) complexes (blue).

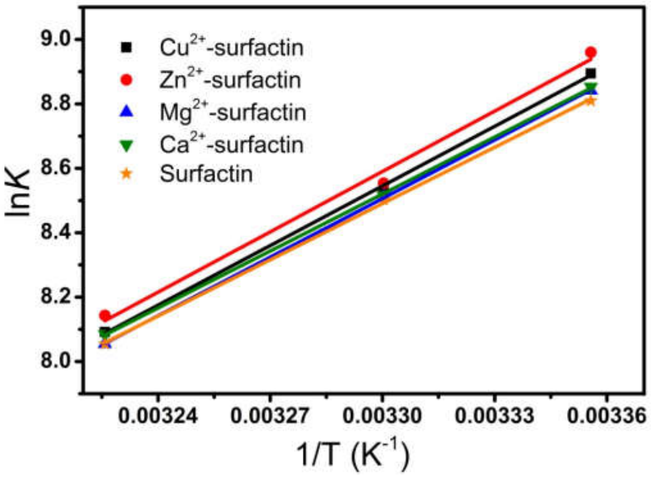

Figure S2. Van't Hoff plot for the interaction of BSA and surfactin or metal-surfactin complexes at different temperatures (298, 303 and 310 K).

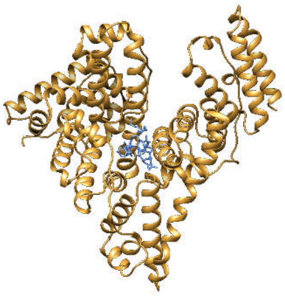

(**a**)

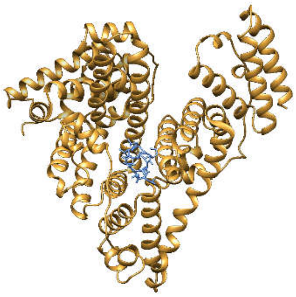

(**b**)

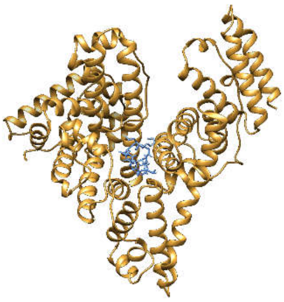

(**c**)

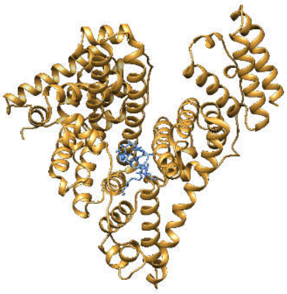

(**d**)

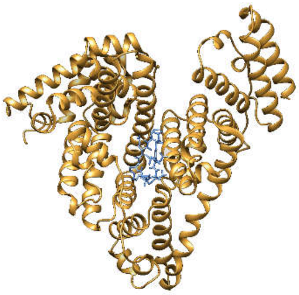

(**e**)

Figure S3. Bovine serum albumin (BSA) in complex with Cu^{2+}-surfactin/BSA (a), Zn^{2+}-surfactin/BSA (b), Mg^{2+}-surfactin/BSA (c), Ca^{2+}-surfactin/BSA (d), and surfactin/BSA (e). BSA was represented by a ribbon structure and investigated complexes were represented by stick model.

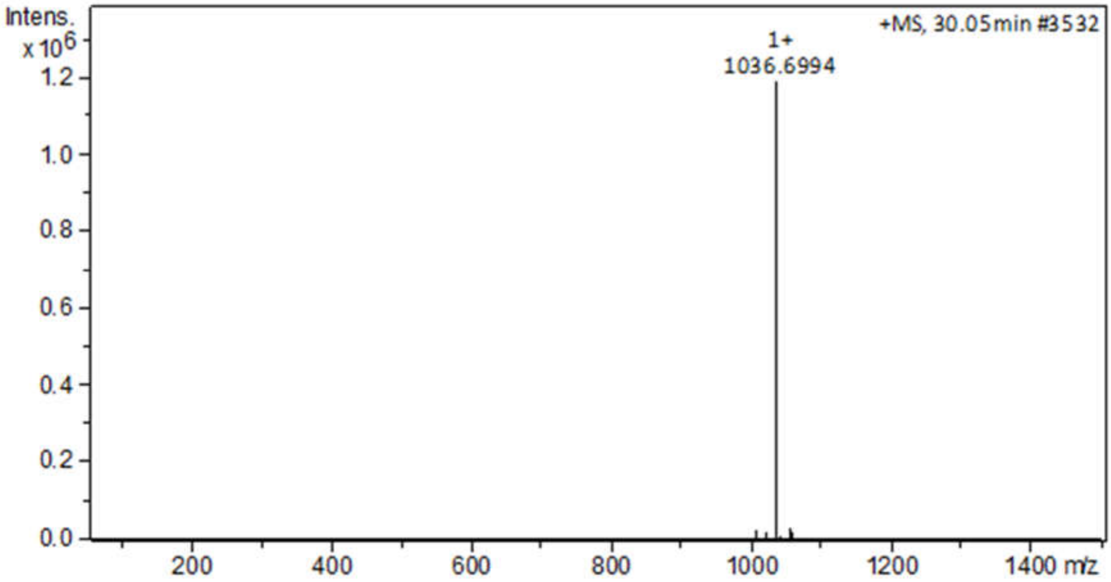

Figure S4. Positive electrospray ionization mass spectrometry (ESI-MS) spectroscopy of surfactin-C_{15}.
